# Prevalence and associated factors of poor sleep quality among Chinese older adults living in a rural area: a population-based study

**DOI:** 10.1007/s40520-019-01171-0

**Published:** 2019-03-27

**Authors:** Peng Wang, Lin Song, Kaili Wang, Xiaolei Han, Lin Cong, Yongxiang Wang, Lei Zhang, Zhongrui Yan, Shi Tang, Yifeng Du

**Affiliations:** 1 Department of Neurology, Shandong Provincial Hospital affiliated to Shandong University, No. 324 Jingwuweiqi Road, Jinan, 250021 Shandong People’s Republic of China; 2 Department of Neurology, Jining No. 1 People’s Hospital, Jining, Shandong People’s Republic of China; 3 Department of Traditional Chinese Medicine, Jining No. 1 People’s Hospital, Jining, Shandong People’s Republic of China

**Keywords:** Poor sleep quality, Pittsburgh Sleep Quality Index (PSQI), Elderly population

## Abstract

**Objective:**

To investigate the prevalence and associated factors of poor sleep quality among community-dwelling elderly population in a rural area of Northern China.

**Methods:**

We conducted a cross-sectional survey in August–December 2014 and recruited 2195 participants who were aged 65 years or older and living in Yanlou Town of Yanggu County in western Shandong Province, China. Data on demographics, health-related behaviors, and clinical conditions were collected through structured interviews. The Pittsburgh Sleep Quality Index (PSQI) was used to assess the sleep quality and patterns. Poor sleep quality was defined as a PSQI score > 7. We employed multiple logistic models to relate poor sleep quality to various factors.

**Results:**

The overall prevalence rates of poor sleep quality were 33.8% in the total sample, 39.2% in women and 26.3% in men (*P* < 0.01). The most common abnormal sleep domains were prolonged sleep latency (39.7%), decreased sleep duration (31.0%), and reduced habitual sleep efficiency (28.8%). Multiple logistic regression analyses revealed that poor sleep quality was significantly associated with female sex (OR = 1.76, 95% CI 1.46–2.12) and clinical comorbidities such as hypertension (OR = 1.28, 95% CI 1.06–1.54), coronary heart disease (OR = 1.60, 95% CI 1.27–2.00), and chronic obstructive pulmonary disease (OR = 1.82, 95% CI 1.34–2.49).

**Conclusions:**

The sleep disorders were highly prevalent among the elderly in rural China. Modifiable risk factors such as cardiometabolic risk factors and disorders were associated with poor sleep quality, which might be potential targets for interventions to improve sleep quality in elderly population.

## Introduction

In 2017, the number of people aged 60 years and above had reached nearly 241 million in China, accounting for 17.3% of the nation’s total population [1]. Large-scale population-based epidemiologic studies have demonstrated that sleep complaints are common in the elderly. For example, the Survey of Health Ageing and Retirement in Europe (SHARE) of people aged 50 years and older from 16 European countries showed that the prevalence of sleep problems varied from ∼ 17% in Denmark and Italy to ∼ 31% in Poland [2]. Furthermore, the US National Health Aging Trends Study (NHATS) found that 28% of the study participants (age ≥ 65 years) experienced poor sleep quality [3]. The French Three-City Study of 9294 community-dwelling persons aged 65 years or older found that 30.9% of older adults suffered from insomnia complaints [4]. In addition, the prevalence of sleep disorders among residents aged ≥ 60 years in northern and southern Chinese urban areas was 37.8% and 41.5%, respectively [5, 6]. Chronic sleep disorders were associated with poor health, and older people with sleep disorders are at marked risk of falls [7], disability [8], hospitalization [9], and mortality [10]. However, the prevalence data of sleep disorders among the elderly in rural areas of northern China are still lacking.

Evidence is emerging that the socio-demographic and lifestyle factors may play a role in sleep disorders among older adults. For instance, female gender, low education, divorce and widowhood, living alone, inadequate fruit intake, drinking tea, alcohol consumption, caffeine, use of certain medications, poor mental health, and physical inactivity have been reported to be associated with sleep disturbance [5, 6, 11–20]. In addition, poor sleep quality or sleep disorders in old people are associated with highly prevalent chronic health conditions, such as hypertension [21], type 2 diabetes [22], cardiovascular disease [23], stroke [24], depression [25], and cognitive impairment [26]. Most of these previous studies have been conducted in western societies. However, the socio-demographic, lifestyle, and medical factors of sleep disorders may differ across ethnic populations. Identifying potentially modifiable factors that are related to poor sleep quality or sleep disorders may help develop effective interventions to improve sleep quality and, thus reduce the risk of chronic diseases in older adults associated with sleep disorders [27].

Therefore, in this large-scale population-based study, we sought to investigate the prevalence of poor sleep quality and abnormal sleep patterns, and to further analyze a range of various factors related to poor sleep quality among older adults who were living in a rural area in western Shandong province, China.

## Methods

### Study population

Participants in this cross-sectional survey were from the Shandong Yanggu Study of aging and health that targeted elderly residents (age ≥ 65 years) who were living in the rural area of Yanlou Town of Yanggu County in western Shandong province, China. From August through December 2014, 2986 (90.7%) of the 3298 eligible participants were recruited. Of these persons, 791 were excluded due to missing data on sleep and other variables, leaving 2195 persons for the current analysis.

### Data collection

Data were collected through face-to-face interviews by trained staff following a structured questionnaire. We collected data on socio-demographics (e.g., age, sex, education, marital status, and occupation), health-related behavioral factors (e.g., smoking and alcohol drinking), and health history [e.g., a physician’s diagnosis of hypertension, diabetes, hyperlipidemia, coronary heart disease (CHD), chronic obstructive pulmonary disease (COPD), and stroke]. Education was divided into illiteracy, primary school (1–5 years), and middle school and above (≥ 6 years). Occupation was dichotomized into farmers vs. non-farmers. We categorized smoking status and alcohol consumption as current vs. non-current (never or former). Marital status was divided into married, unmarried, divorced, and widowed. History of each of those disorders was dichotomized as yes vs. no according to self-reported information.

### Assessments of sleep quality

Sleep quality was measured with the Pittsburgh Sleep Quality Index (PSQI), which is a validated self-rated questionnaire that assesses sleep quality and sleep disturbance over a 1-month period [28]. PSQI includes 19 items that assess seven domains: subjective sleep quality, sleep latency, sleep duration, habitual sleep efficiency, sleep disturbances, use of sleep medication, and daytime dysfunction over the last month. Score for each domain ranges from 0 to 3 (no difficulty to severe difficulty). Scores for all seven domains are summed up to yield a global PSQI score, ranging from 0 to 21. Higher scores indicate worse sleep quality. We defined poor sleep quality as the overall PSQI score > 7 [29]. The abnormal sleep in specific domain was defined as the score for that domain ≥ 2. The Chinese version of the PSQI had good overall reliability (*r* = 0.82–0.83) and test–retest reliability (*r* = 0.77–0.85) [30].

### Statistical analysis

We presented the sociodemographic characteristics, behavioral factors, and clinical conditions of participants by sleep disorders, and tested the statistical differences using the Chi square test for categorical variables and the *t* test for normal distributed continuous variables. We employed logistic regression models to estimate the odds ratios and 95% confidence intervals of sleep disturbance associated with different factors, while adjusting for different potential confounding factors. We reported results from three models: model 1 was controlled for basic demographic factors (age, sex, and education); model 2 was additionally controlled for marital status, occupation, health-related behavioral factors (smoking and alcohol consumption), and clinical conditions (e.g., history of hypertension, hyperlipidaemia, diabetes, CHD, COPD, and stroke); and model 3 represented the best-fit model using the stepwise forward approaches. We considered two-tailed *p* level ≤0.05 to be statistically significant. IBM SPSS Statistics for Windows (version 24.0) (Armonk, NY, USA: IBM Corp) was used for all statistical analyses.

## Results

### Characteristics of study participants

Table 1 shows characteristics of the study participants by sleep quality (global PSQI score > 7 vs. ≤ 7). Of the 2195 participants, the mean age was 71.67 years (SD, 5.89) and 57.9% were women. Overall, the prevalence of poor sleep quality was 33.8% in the total sample, 26.3% in men, and 39.2 in women (for sex difference, *p* < 0.01). Poor sleep quality was significantly (*p* < 0.05) or marginally (0.05 < *p* < 0.07) associated with all the examined factors in Table 1.

**Table 1:** Characteristics of the study participants by sleep quality (*n* = 2195)

Characteristics	Total	Poor sleep quality (PSQI score > 7)		
	Sample	No	Yes	*p* value
No. of subjects	2195	1454 (66.2)	741 (33.8)	
Male	924	681 (73.7)	243 (26.3)	< 0.001
Age (years), mean (SD)	71.67 (5.89)	71.46 (5.69)	72.08 (6.24)	< 0.020
Marital status, married	1580	1065 (73.2)	515 (69.5)	0.065
Occupation, farmers	1985	1305 (89.8)	680 (91.8)	0.129
Education				
Illiteracy	1080	680 (46.8)	400 (54.0)	< 0.001
Primary school	740	493 (33.9)	247 (33.3)	
Middle school or above	375	281 (19.3)	94 (12.7)	
Current smoking	793	563 (38.7)	230 (31.0)	< 0.001
Current alcohol drinking	771	552 (38.0)	219 (29.6)	< 0.001
Hypertension	886	544 (37.4)	342 (46.2)	< 0.001
Hyperlipidemia	363	221 (15.2)	142 (19.2)	< 0.018
Diabetes	188	113 (7.8)	75 (10.1)	< 0.063
CHD	421	229 (15.7)	192 (25.9)	< 0.001
COPD	188	98 (6.7)	90 (12.1)	< 0.001
Stroke	232	141 (9.7)	91 (12.3)	0.063

Data are *n* (%), unless otherwise specified

*PSQI* Pittsburgh Sleep Quality Index, *CHD* coronary heart disease, *COPD* chronic obstructive pulmonary disease

### Distribution of poor sleep quality and subdomains

Table 2 shows the distribution of abnormal Pittsburgh subdomains by age groups and sex. The prevalence rates of abnormal specific sleep domains were 39.7% for sleep latency, 31.0% for sleep duration, 28.8% for habitual sleep efficiency, 26.8% for daytime dysfunction, 23.2% for sleep disturbance, 18.0% for subjective sleep quality, and 4.5% for sleep medicine use. There was no significant difference in prevalence of the abnormal Pittsburgh sleep subdomains among various age subgroups, except habitual sleep efficiency that the prevalence of abnormal habitual sleep efficiency increased with increasing age (*p* < 0.001). Moreover, the prevalence of abnormal PSQI components was higher in women than in men in all sleep subdomains (Table 2).

**Table 2:** Distributions of abnormal Pittsburgh Sleep Quality Index subdomains by age and sex

Age or sex	No. of participants	Abnormal Pittsburgh Sleep Quality Index subdomains^a^, *n* (%)						
		Subjective sleep quality	Sleep latency	Sleep duration	Sleep efficiency	Sleep disturbance	Use of sleep medications	Daytime dysfunction
Total sample	2195	395 (18.0)	871 (39.7)	680 (31.0)	633 (28.8)	510 (23.2)	98 (4.5)	589 (26.8)
Age group, years								
65–69	1005	172 (17.1)	390 (38.8)	322 (32.0)	274 (27.3)	240 (23.9)	44 (4.4)	276 (27.5)
70–74	581	106 (18.2)	226 (38.9)	166 (28.6)	148 (25.5)	131 (22.5)	31 (5.3)	151 (26.0)
75–79	332	60 (18.1)	128 (38.6)	105 (31.6)	107 (32.2)	73 (22.0)	12 (3.6)	86 (25.9)
≥ 80	277	57 (20.6)	127 (45.8)	87 (31.4)	104 (37.3)	66 (23.8)	11 (4.0)	76 (27.4)
*p* value		0.614	0.168	0.533	0.001	0.865	0.621	0.894
Sex								
Male	924	123 (13.3)	280 (30.3)	249 (26.9)	216 (23.4)	200 (21.6)	34 (3.7)	230 (24.9)
Female	1271	272 (21.4)	591 (46.5)	431 (33.9)	417 (32.8)	310 (24.4)	64 (5.0)	359 (28.2)
*p* value		< 0.001	< 0.001	< 0.001	< 0.001	0.133	0.129	0.080

^a^Score for each domain ranges from 0 to 3 (no difficulty to severe difficulty), and a domain score ≥ 2 indicates abnormal sleep in the domain

### Associated factors of poor sleep quality

Table 3 shows the association of poor sleep quality (defined as a PSQI score > 7) with different demographic, behavioral, and clinical factors. When controlling for demographic factors, poor sleep quality was significantly associated with female sex, current smoking, hypertension, hyperlipidemia, CHD, COPD, and stroke (Table 3, model 1). When entering all variables examined in Table 3 into the model simultaneously, the associations of current smoking, hyperlipidemia, and stroke with poor sleep quality were diluted and became statistically non-significant (Table 3, model 2). We detected a marginally statistical interaction between gender and smoking on sleep disorders (*p* for interaction = 0.10). Stratifying analysis by gender showed that the age- and education-adjusted odds ratio of sleep disorders was 1.61 (95% CI 1.08–2.41) in men and 1.30 (0.73–2.31) in women, and the multiple-adjusted odds ratio was 1.40 (0.90–2.17) in men and 1.18 (0.61–2.26) in women. Finally, when using the forward stepwise multivariate analysis to fit the best model, the following factors showed significant associations with poor sleep quality: female sex (OR = 1.76, 95% CI 1.46–2.12), hypertension (OR = 1.28, 95% CI 1.06–1.54), CHD (OR = 1.60, 95% CI 1.27–2.00), and COPD (OR = 1.82, 95% CI 1.34–2.49) (Table 3, model 3).

**Table 3:** Odds ratio (95% confidence interval) of poor sleep quality associated with demographic, behavioral, and clinical factors

Factors	Model 1^a^		Model 2^a^		Model 3^a^	
	Odds ratio (95% CI)	*p*	Odds ratio (95% CI)	*p*	Odds ratio (95% CI)	*p*
Sex (female vs. male)	1.72 (1.39–2.13)	< 0.001	2.09 (1.47–2.96)	< 0.001	1.76 (1.46–2.12)	< 0.001
Age (years)	1.07 (0.98–1.17)	0.090	1.09 (0.99–1.19)	0.064	–	–
Education (years)	0.94 (0.82–1.09)	0.471	0.92 (0.79–1.07)	0.287	–	–
Occupation (farmers vs. non-farmers)	1.05 (0.75–1.47)	0.757	1.02 (0.72–1.44)	0.897	–	–
Marital status (married vs. others)	1.00 (0.81–1.24)	0.937	0.97 (0.78–1.21)	0.848	–	–
Current smoking	1.50 (1.08–2.08)	0.014	1.35 (0.94–1.93)	0.096	–	–
Current alcohol drinking	1.11 (0.85–1.44)	0.445	0.97 (0.72–1.31)	0.887	–	–
Hypertension	1.39 (1.16–1.66)	< 0.001	1.23 (1.01–1.49)	0.035	1.28 (1.06–1.54)	0.008
Hyperlipidemia	1.32 (1.04–1.68)	0.020	1.03 (0.80–1.34)	0.789	–	–
Diabetes	1.31 (0.96–1.79)	0.086	1.14 (0.82–1.58)	0.416	–	–
Coronary heart disease	1.81 (1.45–2.25)	< 0.001	1.58 (1.25–1.99)	< 0.001	1.60 (1.27–2.00)	< 0.001
COPD	1.99 (1.47–2.71)	< 0.001	1.78 (1.30–2.44)	< 0.001	1.82 (1.34–2.49)	< 0.001
Stroke	1.44 (1.08–1.92)	0.011	1.29 (0.96–1.74)	0.090	–	–

*COPD* chronic obstructive pulmonary disease

^a^Odds ratios (95% confidence intervals, CIs) in Model 1 were derived from the logistic regression model that was adjusted for age, sex, and education, odds ratios (95% CIs) in Model 2 were derived from the logistic regression model that included all these factors in the table, and odds ratios (95% CIs) in Model 3 were derived from the best-fit model using the forward stepwise approaches

## Discussion

In this large-scale population-based study, we showed that poor sleep quality affected nearly one-third of older adults who were living in the rural communities in China, which was slightly higher than that of those living in the urban areas [5, 6]. The prevalence rates of sleep disturbance in the present study were 26.3% in men and 39.2% in women. These results are in contrast to some of the previous studies from urban areas, where elderly men had a higher prevalence of sleep disturbance than that of elderly women in China [5, 6]. Of note, more than 80% of our study population had no formal education or achieved primary school culture, and the majority of them were farmers, and about one-third had smoking and alcohol drinking hobbies, which might partly contribute to the higher prevalence rate in rural older adults, because research has indicated that rural people may ignore the importance of a healthy lifestyle and high quality sleep [29]. Among the seven PSQI domains, sleep latency was most highly prevalent (39.7%), followed by sleep duration (31.0%) and habitual sleep efficiency (28.8%). These results were slightly different from those of other rural adults or the elderly in other provinces in China [29, 31]. Variations in diagnostic criteria, interview techniques, and age of the study populations may also partly contribute to the inconsistent results across studies.

In the present study, we found that female gender was associated with an increased likelihood of poor sleep quality, which is in line with the current view that women are more prone than men to experiencing sleep disorders [32–34]. The epidemiologic studies have indeed frequently indicated that female gender is an independent risk factor for sleep disorders [35, 36]. The reason for poor sleep in women may be due to the fact that compared to men, women had higher proportions of low education, low individual income, and more chronic diseases, and were more susceptible to depression and anxiety [5, 31, 37].

In this study, suffering from some chronic diseases was associated with poor sleep quality, even after controlling for multiple potential confounders, such as hypertension, CHD, and COPD. A meta-analysis of 17 prospective cohort studies showed that short sleep duration, sleep continuity disturbance (SCD), early-morning awakening (EMA) and combined symptoms of insomnia were associated with an increased risk of hypertension [38]. Sleep-disordered breathing has been associated with chronically elevated blood pressure and hypertension in the US Wisconsin Sleep Cohort Study [39]. A systematic review and meta-analysis of prospective cohort studies indicated that compared with a sleep duration of 7 h per day, the risk of CHD increases by 11% per 1-h decrease in sleep duration and increases by 7% per 1-h increase in sleep duration [40]. Moreover, a cohort study showed that participants with untreated severe sleep-disordered breathing (AHI > 30) were 2.6 times more likely to develop CHD or heart failure compared to those without sleep-disordered breathing [41]. A previous study showed that patients with higher COPD assessment test (CAT) scores generally had more severe insomnia [42]. Nighttime symptoms are usually common among patients with COPD, including trouble falling asleep, experienced nighttime awakenings, trouble staying asleep, and woke after a usual amount of sleep feeling worn out [43].

Several potential mechanisms might explain the potential link of sleep disorders with chronic diseases. First, the common mechanism connecting sleep disorders or poor sleep quality to the development of hypertension is that alterations in sleep quality/quantity lead to the loss of the nocturnal dip in blood pressure (BP) that is associated with non-rapid eye movement (NREM) sleep as the first step toward the development of hypertension. Further, loss of the BP dipping pattern is mainly attributable to an increase in nocturnal sympathetic activity, which in turn leads to a diurnally permanent increase in sympathetic tone, eventually mediates the occurrence of hypertension [44]. Second, abnormal sleep may lead to weight gain, metabolic disorders, inflammation, sympathetic activation, high blood clotting and endothelial dependent vasodilator dysfunction [40, 45]. Sleep-disordered breathing may cause oxidative stress, sympathetic activation, inflammation, hypercoagulability, endothelial dysfunction, and metabolic deregulation [46]. These can be linked with CHD. Third, patients with COPD are often accompanied by inadequate pulmonary ventilation and hypoventilation during sleep, then will lead to hypoxemia and hypercapnia, which have been associated with increased frequency of arousals during sleep [47]. Moreover, cough and wheeze [48], inhaled corticosteroid, and phlegm production are also associated with poor sleep quality [49].

Our population-based study targeted older residents living in less developed rural areas in China where little attention has been paid thus far by the research communities. Further, we used the internationally recognized and also validated scale in Chinese population to assess sleep quality and patterns. However, our study also has limitations. First, the cross-sectional nature of our study limited the possibility to make the causal inference. Second, lifestyle factors and medical disorders were assessed based on self-reported data, which might be subject to information bias. We ascertained the data from detailed questionnaire of health history including lifestyle factors, the time of first diagnosis and medication use of chronic diseases. Third, despite the adjustment for multiple potential confounding factors, other unknown or unmeasured confounding variables, such as physical activity [50], coffee or tea consumption, anxiety, and depression were not available [51].

In summary, poor sleep quality or sleep disorders are highly prevalent in a rural elderly population in China. In addition, some manageable behavioral factors and chronic health conditions were associated with a poor sleep quality. Potential influencing factors and the comprehensive intervention for sleep disorders are important topics for future research.
